# Chronic heavy alcohol intake and liver‐specific LRP‐1 reduction increase amyloidosis in Alzheimer’s disease mice

**DOI:** 10.1002/alz.087662

**Published:** 2025-01-03

**Authors:** Devaraj V. Chandrashekar, G. Chuli Roules, Ross A. Steinberg, Urvashi R. Panchal, Nataraj Jagadeesan, Adenike Oyegbesan, Trinh Roselyn, Joshua Yang, Sharda R. Bhagwat, Hiya Rakholia, Rutvi D. Mevawala, Moom Roosan, Derick Han, Rachita K. Sumbria

**Affiliations:** ^1^ School of Pharmacy, Chapman University, Irvine, CA USA; ^2^ Keck Graduate Institute, Claremont, CA USA; ^3^ The Millennium School, Surat, Gujarat India

## Abstract

**Background:**

Chronic heavy alcohol drinking may be a modifiable risk factor for Alzheimer’s disease (AD), but studies in rodent AD models more closely mimic chronic moderate alcohol drinking in humans and largely focus on the brain. The role of the liver, which is significantly impacted by chronic heavy alcohol intake, in driving brain changes in alcohol‐dependent AD remains unexplored. Our study using intragastric‐ethanol feeding, which mimics chronic heavy alcohol intake in humans, in C57BL/6J mice showed significant AD‐relevant changes in the brain and liver. Therefore, we aimed to investigate how hepatic changes using this model of chronic heavy drinking drive AD pathology in AD mice, which has never been attempted.

**Methods:**

Eight‐month‐old male APP/PS1 mice were fed ethanol or control diet intragastrically for 5 weeks (n = 7‐11/group). Brain and liver Aβ were assessed using immunoassays. Three important mechanisms of brain amyloidosis were investigated: hepatic LRP‐1 (major peripheral Aβ regulator), blood‐brain barrier (BBB) function (vascular Aβ regulator), and microglia (major brain Aβ regulator) using immunoassays. Hepatic LRP‐1 expression was confirmed using Nanostring spatial transcriptomics. To elucidate the role of hepatic LRP‐1 in brain amyloidosis, hepatic LRP‐1 was silenced by injecting LRP‐1 microRNA delivered by the adeno‐associated virus 8 (AAV8) and the hepato‐specific thyroxine‐binding globulin promoter to 4‐month‐old male APP/PS1 mice (n = 6). Control APP/PS1 mice received control AAV8 (n = 6). Spatial memory was assessed 12 weeks after LRP‐1 silencing using Y‐maze, and brains and livers were harvested to detect Aβ.

**Results:**

Alcohol feeding increased aggregated Aβ (p<0.05) by ELISA and 6E10‐positive Aβ load (p<0.05) by immunostaining, and reduced plaque‐associated microglia in APP/PS1 mice brains. Further, alcohol‐fed APP/PS1 had liver steatosis and significantly downregulated hepatic LRP‐1 (p<0.01) at the protein and transcript level, and brain and hepatic Aβ were positively correlated (p<0.05). Hepato‐specific LRP‐1 silencing significantly increased brain Aβ load (p<0.05) and reduced entries into the novel arm of the Y‐maze (p<0.05) in APP/PS1 mice.

**Conclusion:**

Chronic heavy alcohol intake reduced hepatic LRP‐1 expression, and hepato‐specific LRP‐1 silencing increased brain Aβ and spatial memory deficits in APP/PS1 mice. Our results place hepatic LRP‐1 as a potential key driver of brain amyloidosis in alcohol‐dependent AD.

